# Management and Followup of Complicated Crown Fractures in Young Patients Treated with Partial Pulpotomy

**DOI:** 10.1155/2013/597563

**Published:** 2013-06-20

**Authors:** Francisco Ojeda-Gutierrez, Brenda Martinez-Marquez, Soraya Arteaga-Larios, M. Socorro Ruiz-Rodriguez, Amaury Pozos-Guillen

**Affiliations:** ^1^General Dentistry Department, Facultad de Estomatología, Universidad Autónoma de San Luis Potosí, 2 Dr. Manuel Nava, Zona Universitaria, 78290 San Luis Potosí, SLP, Mexico; ^2^Pediatric Dentistry Posgraduate Program, Facultad de Estomatología, Universidad Autónoma de San Luis Potosí, 2 Dr. Manuel Nava, Zona Universitaria, 78290 San Luis Potosí, SLP, Mexico

## Abstract

Two cases of young patients with traumatized permanent teeth having complicated crown fractures are reported. Endodontic management included partial pulpotomy by the Cvek technique; restorative management included resin restoration and reattachment of the teeth fragments. Treatments were considered successful in all cases according to the following criteria: absence of clinical symptoms, absence of X-ray signs of pathology, and presence of pulpal vitality 6 to 25 months after treatment.

## 1. Introduction 

Trauma to the facial area represents a public health problem involving children and adolescents; it generally involves the teeth and their supporting structures. The most frequent causes are falls, traffic accidents, domestic violence, fights, and sports. Most dental injuries occur during the first 2 decades of life, especially between 2 and 3 years and between 8 and 12 years of age, occurring more often in boys than in girls [1–3]. Dental fractures frequently involve only the enamel, or enamel and dentin, without affecting the pulp [4]. Occasionally, however, the pulp is also involved [5–7]. Due to their position, the teeth most frequently affected by dental traumatism are the maxillary incisors: 80% centrals and 16% laterals [8]. Several diagnostic criteria have been used to classify traumatic dental injuries. Ellis and Davery [9], proposed a classification based on a numerical system (I to VIII) and described the extent, using terms like “simple fracture” and “complicated fracture”; this classification considers X-ray examinations and vitality tests. 

Treatment of crown fractures with exposed pulp in permanent young teeth depends on the degree of pulp exposure, time between accident and examination, effect of the traumatism, and the stage of root development. Treatment options of crown fractures with pulpal exposure are direct pulp capping, partial pulpotomy, pulpectomy, or extraction. For young patients in whom the exposed pulp maintains its vitality, pulpotomy is the best endodontic treatment option in order to maintain pulpal functions [10–13]. A partial pulpotomy, known as the Cvek technique, is indicated for teeth having the following characteristics: small pulp exposure, treated within 14 days of trauma, caries-free, open apex or thin dentinal walls, and vital and asymptomatic pulp. This technique involves amputation of the pulp 2 mm apical to the affected pulp tissue, but it is not recommended for those cases in which the pulp exposure is extensive or where there has been a 2-week lapse between trauma and treatment [14]. The aim of the present report is to describe the management and followup of 2 cases of dental trauma with complicated crown fractures who were treated by partial pulpotomy using the Cvek technique. 

## 2. Case Presentation

### 2.1. Case 1

**Figure 1:**
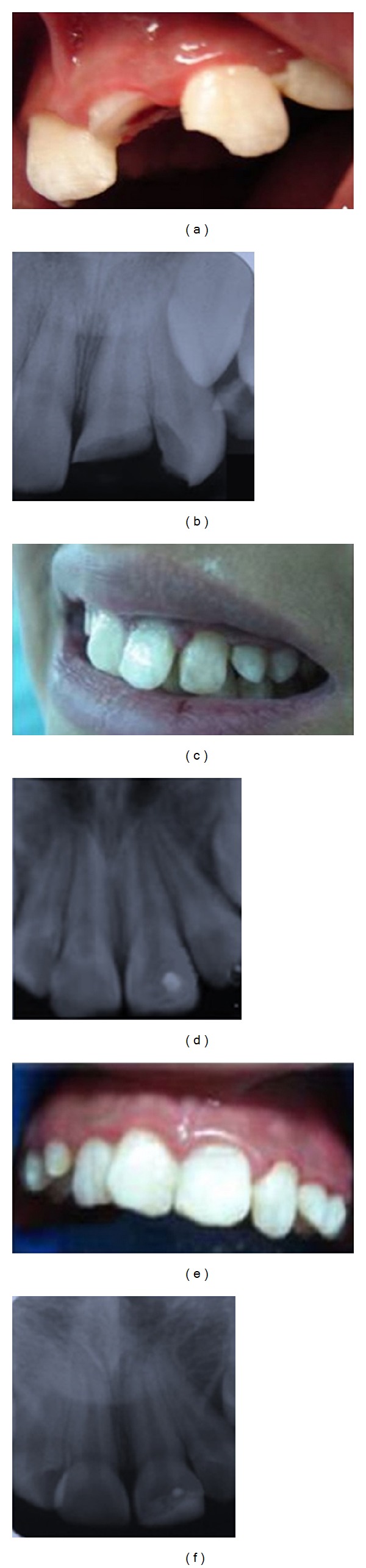
(a) Initial clinical image of patient 4 h after trauma with complicated crown fracture in maxillary left central incisor, with ulcerated and exposed pulp, and enamel crown fracture in left lateral incisor. (b) Initial radiograph showing loss of dental structure, complete root development, closed apices, normal periodontal ligament, and absence of root or alveolar bone fractures. (c) Resin reconstruction and reattachment of tooth fragments after treatment. (d) Posttreatment radiograph showing indirect pulp capping with resin reconstruction in the maxillary left lateral incisor and pulpotomy with reattachment of the dental fragments in the left central incisor. (e-f) Clinical and radiographic examinations 6 months after trauma. Patient showed no periodontal or periapical pathology, nor pulpal signs or symptoms.

An 11-year-old boy was referred to our clinic because of crown fractures of the maxillary left central and lateral incisors, presenting to the clinic 4 hours after the trauma. According to his medical history, the patient exhibited neither systemic disease nor relevant problems. Extraorally, there was no apparent trauma to the soft tissues. Intraoral clinical examination revealed a complicated crown fracture of the maxillary left central incisor (class III, Ellis's classification), with ulcerated and exposed pulp, and extensive crown fracture with noticeable dentinal involvement. There was no pulp exposure of the left lateral incisor (class II, Ellis's classification) (Figure 1(a)). For both teeth, periapical radiographic examination showed complete root development, closed apices, no periapical injury, and no alveolar bone fractures (Figure 1(b)). Endodontic treatment included pulpal protection with glass ionomer and for the left lateral incisor, reconstruction with hybrid resin, and partial pulpotomy using the Cvek technique with reattaching of the same teeth fragments for the central incisor. The treatment plan was accepted. 

A local anesthetic was administered and the affected teeth were isolated with a rubber dam. For indirect pulp protection in the lateral incisor, a layer of glass ionomer (Vitrebond; 3 M ESPE, St Paul, MN, USA) was applied. Then the tooth was acid-etched using 37% orthophosphoric acid for 30 s, and the acid was eliminated by rinsing with distilled water and drying; dental adhesive (Prime and Bond NT, Dentsply Caulk, Milford, DE, USA) was applied according to the manufacturer's instructions. A hybrid resin (Z-250, 3 M ESPE) was applied using the incremental technique. Each increment was light cured for 40 s. For the partial pulpotomy of the central incisor, a number. 330 tungsten round bur (with continuous saline rinsing) was used to amputate the pulp close to the exposure site to a depth of 2 mm. The blood was noted to be light red, and hemostasis was evident in 2 min. A dressing of calcium hydroxide (Ca[OH]_2_) paste (Viarden, Mexico City, DF, Mexico) was placed, followed by a coat of glass ionomer (Vitrebond), and photopolymerized for 40 s. The teeth fragments were reattached using a modified Simonsen's technique [7]. Clinical and radiographic examinations were made after treatment (Figures 1(c)-1(d)). Followup appointments took place 1 week, 1 month, and 3 months after treatment, with no pulpal signs or symptoms found. Six months after the trauma, the teeth were found to be vital without periodontal or periapical pathology and the restorations were functional and aesthetically acceptable (Figures 1(e)-1(f)).

### 2.2. Case 2

**Figure 2:**
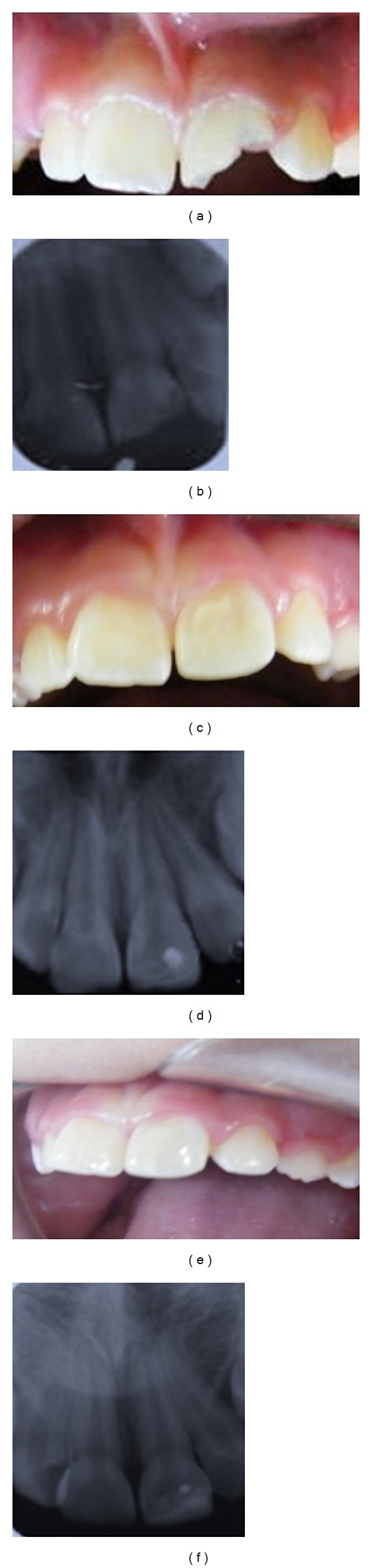
(a) Initial clinical image of patient 17 hours after trauma with complicated crown fracture in the maxillary left central incisor. (b) Initial radiograph showing loss of dental structure, complete root development, closed apices, and absence of root or alveolar bone fractures. (c) Resin reconstruction after treatment. (d) Posttreatment radiograph showing partial pulpotomy with resin reconstruction. (e-f) Clinical and radiographic images 25 months after treatment. Patient's teeth were found to be vital; no pain to percussion or palpation, with functional restorations.

A 9-year-old girl was seen in our clinic with trauma to her maxillary left incisor area, received 17 hours previously. Intraoral examination revealed a complicated crown fracture of the left central incisor (class III, Ellis's classification), with ulcerated pulp (Figure 2(a)). Periapical radiographic examination showed completed root formation, closed apices, no periapical injury, and no alveolar bone or radicular dental fractures (Figure 2(b)). Pulp management included partial pulpotomy with the Cvek technique and reconstruction with hybrid resin of both involved teeth. Endodontic and restorative treatments were realized as in the previously reported case 1 (Figures 2(c)-2(d)). Followup appointments were made periodically. At 25 months' follow-up, the teeth were found to be vital, without periodontal or periapical pathology (Figures 2(e)-2(f)).

## 3. Discussion 

Reports have shown that 25% of school-aged children will experience some kind of dental trauma [15]. Among the child and teenage population, the possibility of suffering orofacial trauma is high and actually is considered a dental public health problem [16]. Crown fractures with pulp exposure represent 18% to 20% of traumatic injuries involving the teeth, the majority being in young permanent teeth [6]. Complicated crown fractures are defined as fractures involving enamel and dentin with pulp exposure. These injuries produce changes in the exposed pulp tissues, and a biological and functional restoration represents an important clinical challenge. In these cases, inflammation or contamination is generally present.

For traumatized teeth with complicated crown fractures in young patients, treatment options include direct pulp capping, partial pulpotomy, cervical pulpotomy, pulpectomy, or extraction, depending on the time between the trauma and treatment of the patient, degree of root development, and size of the pulp exposure. Pulp exposure caused by dental trauma has a better prognosis because of the absence of microorganisms associated with caries. The objective is always to preserve pulp vitality. Pulp capping is recommended for small exposures (1 mm) that have occurred not more than a few hours previously [17]. Partial pulpotomy may be the preferred treatment in cases of extensive pulpal exposure when pulpal vitality and the time elapsed between trauma and treatment allow for this option. A pulpotomy is indicated for those patients wherein the pulpitis has not progressed beyond the coronal pulp, bleeding after amputation is not excessive, and the blood has a normal color [14, 18].

Recently Andreasen et al. [19] estimated that 2 out of 3 children suffer a traumatic dental injury before adulthood and established that the problem of trauma in children is not reflected by the active participation of pediatric dentists in acute treatment, followup, or research on this topic. 

 Partial pulpotomy has a high success rate in cases with complicated crown fractures in young teeth with pulp exposure [20–24]; however, a long-term followup is necessary to establish this success rate. Cvek reported high success rates in cases of complicated crown fractures treated by partial pulpotomy (96%) having a followup of between 14 and 60 months and 30 hours between trauma and treatment [14]. Partial pulpotomy has the advantage of preserving the cell-rich coronal pulp tissue, which possesses better healing potential and can maintain the physiologic deposition of dentin [20]. Various materials have been proposed as medicaments for pulpotomies such as mineral trioxide aggregate (MTA) and enamel matrix derivative (EMD) [25–27]. MTA's effects on amputated pulpal tissue suggest that the material preserves the pulp tissue and promotes the regeneration of hard tissues [28]. EMD, because of its amelogenin and amelin protein-rich fraction, has the potential to induce a process that seems to imitate normal dentinogenesis; it clearly influences the odontoblasts and endothelial cells of the pulp capillaries to create a hard, calcified barrier over the pulp exposure [27, 29, 30]. However; due to its action, Ca(OH)_2_ continues to be the material of choice for pulpotomy [6, 7, 23, 24, 31]. This agent prevents bacterial activity and stimulates dentin bridge formation. Its high pH and low water solubility are responsible for its antimicrobial activity and ability to induce hard tissue formation.

 The followup for determining the success of treatment is based on clinical and radiographic evaluations. During followup of the 2 cases reported at different periods (6−25 months), no tooth sensitivity or pain was registered; also, no symptoms or radiographic defects were present. Clinical and radiographic examination showed no periodontal or periapical pathology, and the restorations were functionally acceptable and aesthetically gratifying. In the present cases, we decided to use a partial pulpotomy on the affected teeth. For this decision we considered the size of the exposure, interval between the accident and treatment, age of the patient, and maturity of the roots. During followups, we evaluated the vitality of the pulp. The potential for the pulp to recover its vitality depends on several factors such as the state of the pulpal tissue before the trauma, previous inflammation, infection associated with the caries, and the treatment [17, 32–34]. The patients showed no periodontal or periapical pathology, pulpal signs or symptoms, mobility, color changes, edema, fistula, calcification of the root canal, or alterations in the apical region. Another clinical criterion of success is the capacity of the pulp to recover its vitality [35, 36]. The teeth treated in the 2 reported cases were found to be vital, having the formation of dentinal bridges and continuous root development. The treatments substantiated the effectiveness of this pulp procedure, within a postoperative observation time of 6 to 25 months. 

Partial pulpotomy represents an excellent alternative for the treatment of traumatized vital teeth. On the basis of these reports, we recommend the partial pulpotomy using the Cvek technique for traumatized teeth with complicated crown fractures.
